# Tocochromanols and Chlorophylls Accumulation in Young Pomelo (*Citrus maxima*) during Early Fruit Development

**DOI:** 10.3390/foods10092022

**Published:** 2021-08-28

**Authors:** Yihan Zhao, Junhao Li, Shaohua Huang, Huayong Li, Yutao Liu, Qiuming Gu, Xinbo Guo, Yuwei Hu

**Affiliations:** 1 School of Food Science and Engineering, South China University of Technology, Guangzhou 510640, China; zhaoyihan19980515@163.com; 2 College of Food Science, South China Agricultural University, Guangzhou 510642, China; 201922210107@stu.scau.edu.cn; 3 Key Laboratory of South China Modern Biological Seed Industry, Ministry of Agriculture and Rural Areas, National S&T Innovation Center for Modern Agricultural Industry, Guangzhou 510520, China; kczx_hsh@gd.gov.cn (S.H.); kczx_hy@gd.gov.cn (H.L.); drliuyutao@163.com (Y.L.); 4 Guangdong Lijinyou Agricultural Technology Co., Ltd., Meizhou 514743, China; guqm@163.com

**Keywords:** pomelo, tocochromanol, chlorophyll, biosynthesis, fruit development, by-products

## Abstract

Pomelo is an important cultivar of the genus Citrus that contains a variety of beneficial nutrients, and its young fruit is an agricultural by-product that is currently not fully utilized because it is often thrown away during cultivation and management. In this study, the dynamics of tocochromanol during young pomelo development were investigated by measuring chlorophyll content, tocochromanol accumulation, and expression levels of related genes during early fruit development. The results showed that chlorophyll content decreased overall during these four developmental stages and had some synergism with tocochromanol. Four tocochromanol components were detected in pomelo of both genotypes, and α-tocopherol was the main component. The tocochromanol content of honey pomelo was highest in the first period, reaching 70 ± 5 μg/g in dry weight (DW), and golden pomelo peaked in the second period at 86.10 ± 0.18 μg/g DW, with an overall decreasing trend in both genotypes. The different gene expression patterns of the tocochromanol biosynthesis pathway could partially explain the changes in these components and further elucidate the regulatory mechanisms of tocochromanol accumulation during early fruit development. As a natural product, young pomelo fruit is an attractive source of tocochromanol and has potential application in industrial production. The results of this study may provide directions for the high additional value utilization of young pomelo fruit.

## 1. Introduction

Tocochromanol is considered an effective fat-soluble natural antioxidant, which is synthesized mainly in photosynthetic organisms such as plants, algae, and some cyanobacteria [1]. Studies have found that tocochromanol plays a fundamental biological role in protecting cells and tissues from oxidative damage and other side effects caused by cytotoxic drugs [2]. From the perspective of structure, tocochromanol is a derivative of benzodihydropyran, including tocopherol and tocotrienol. There are eight monomers of tocochromanol, namely α, β, γ, δ-tocopherol and α, β, γ, δ-tocotrienol [3]. Tocopherols widely exist in fruits, leaf chloroplasts, cotyledons, hypocotyls, and stems, and α-tocopherol is the main form of tocochromanol in photosynthetic tissue. However, tocotrienols have a unique distribution in specific tissues, such as seeds and the pericarp [4]. Tocochromanol is synthesized by a polar phenolic moiety, p-hydroxyphenylpyruvic acid (HPP), and a polyprenyl side chain derived from isopentenyl diphosphate (IPP). HPP was from the shikimate pathway. IPP was the precursor of phytyl diphosphate (phytyl-PP, C20) and geranylgeranyl diphosphate (GGDP, C20), which derived side chains of tocopherol and tocotrienol, respectively [5], and made by the methylerythritol phosphate pathway (DOXP) [1].

Each tocochromanol monomer has special functions. Among them, α-tocopherol has the strongest biological activity and is the most widely distributed and abundant tocochromanol form in nature. Some studies have found that the lack of α-tocopherol may affect the oxidation of low-density lipoprotein (LDL) and the progress of atherosclerosis in human physiology [6]. Moreover, α-tocopherol also plays an important role in the anti-cancer effect. Intake of α-tocopherol is beneficial to remove peroxides and superoxides produced during the metabolism of carcinogens, thereby preventing the oxidative damage and malignant transformation of cervical neoplasia [7]. Meanwhile, α-tocopherol inhibited the growth of prostate cancer cells through apoptosis, with daily intake reducing the risk of prostate cancer by 32% in a large controlled clinical trial in Finland [8]. In addition, since several health promotion characteristics of tocotrienol are somewhat different from those of tocopherol [9], it has recently attracted more and more scientific interest. For example, tocotrienol can prevent nerve cells from oxidative damage and can inhibit adhesion molecules, induce cell cycle arrest and down-regulate c-Myc and telomerase [10]. Tocotrienol was also shown to decrease the number of inflammatory cell infiltrations and preserve follicle morphology better following cyclophosphamide chemotherapy [11]. Moreover, endoplasmic reticulum stress and autophagy induced by γ-tocotrienol promote the death of human breast cancer cells through moderate but significant attenuation of tocotrienol-induced cytotoxicity [12].

In recent years, some studies have found that tocochromanol metabolic pathway is closely related to chlorophyll metabolism. Valentin et al. [13] isolated *VTE5* and *slr1652* genes encoding phytosterol kinases from Arabidopsis and algae cells, which are phosphatase genes responsible for converting phytosterol into tocopherol synthesis precursor phytyl diphosphate (PDP). When the *VTE5* gene lost its function, the content of tocopherol in the leaves and seeds of Arabidopsis homozygous mutant *vte5* was only 35% and 20% of that of wild-type seeds, respectively, while the mutation of algal cell *slr1652* resulted in a half decrease in tocopherol content. A large accumulation of phytol was found in both mutants, indicating that chlorophyll decomposition products were the primary substrate for tocopherol synthesis. At the same time, Katharina vom Dorp et al. [14] further found that the main pathway of tocopherol synthesis was the phosphorylation reaction of chlorophyll-decomposing phytol to PDP. Moreover, tocotrienol precursor GGDP is also involved in chlorophyll synthesis [15].

Pomelo is one of the genus Citrus, a dominant agricultural product in China and planted in the vast area. Citrus fruit not only has a unique flavor but also contains a lot of tocochromanol, oligosaccharides and other important nutrients, which can promote human health. It was found that Citrus fruit or juice intake was negatively correlated with several diseases [16]. The objects of this study are honey pomelo (*Citrus grandis* cv. Miyou) (HP) and golden pomelo (*Citrus maxima* (Burm.) Merr. cv. Meizhou Yu) (GP), which are the two most common pomelo genotypes in South China, and also the most productive pomelo genotypes in Guangdong Province. Although golden pomelo and honey pomelo are rich in resources and sell well, the annual shedding of young pomelo fruits causes a significant waste of resources. Rational utilization of tocochromanol in Citrus fruit drop can not only turn waste into treasure and improve the production value of pomelo but also improve the nutritional value of food and play a good health role when the extracted useful substances are added to food.

Studies on Citrus fruits have mainly focused on the determination of flavonoids, ascorbic acid, and phenols [17], but little is known about tocochromanol. This study determined the content and composition of tocochromanol in honey pomelo and golden pomelo at four developmental stages with high-performance liquid chromatography-fluorescence detection (HPLC-FD). It was assumed that tocochromanol accumulation might be different during pomelo development due to changes in gene transcription levels in the biosynthesis pathway. Therefore, this study aimed to investigate the changes of tocochromanol accumulation in pomelo at different development stages to reveal the relationship between tocochromanol and their related gene expression in the biosynthesis pathway during early fruit development.

## 2. Materials and Methods

### 2.1. Plant Materials and Sample Collection

**Figure 1:**
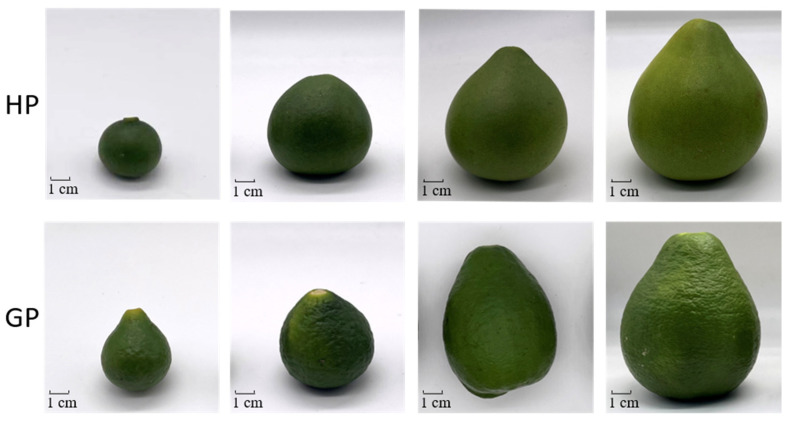
Pictures of pomelo fruit development stages. S1–S4 four sampled stages during fruit development. The upper fruits are “Honey pomelo” (HP) and the down fruits are “Golden pomelo” (GP).

The experimental pomelos were cultivated in Meizhou Pomelo National Industrial Park (Meizhou, China), controlled by an intelligent agricultural monitoring system and collected from late April to early May 2021. Young fruits with uniform color and fullness were selected as samples. All samples from the same developmental period were sampled evenly from three trees approximately 10 years old with the same growth environment to maintain the same genotype. Ten fruits were taken from each tree at the same stage, with 30 fruits at each stage. The collected pomelos were divided into four developmental stages with corresponding diameters of 2 cm, 4 cm, 6 cm, and 8 cm (Figure 1). All fresh young fruits were cleaned and cut into 2–3 mm slices, and three samples were taken evenly for both genotypes and stored at −80 °C for RNA extraction. Then the remaining samples were frozen in liquid nitrogen and dried in a vacuum freeze drier. After that, the samples were crushed with a liquid nitrogen grinder and stored hermetically at −20 °C for tocochromanol extraction and determination.

### 2.2. Extraction and Determination of Tocochromanol

Tocochromanol was extracted from pomelo using the previously reported method [18]. In short, at first frozen pomelo powder was added to 95% ethanol, then 0.3 M sodium chloride, 0.5 M pyrogallol ethanol and 1 M ascorbic acid were successively added for antioxidation and 10.7 M potassium hydroxide was added. After that, mixtures were saponified for 45 min and finally extracted with n-hexane/ethyl acetate (9:1 *v*/*v*). Collect organic layers and dry them with nitrogen gas in a nitrogen blower (Tianjin Hengao Technology, Tianjin, China). After solvent evaporation, the residue was dissolved in n-hexane solution with 1% isopropyl alcohol for analysis.

Tocochromanol was quantified using a previously used high-performance liquid chromatography-fluorescence detection (HPLC-FD) method [19]. The analysis system is equipped with a Waters 515 HPLC pump and a Waters 2475 Multi λ fluorescence detector (Waters Corporation, Milford, MA, USA). The HPLC conditions were: an Agilent ZORBAX RX-SIL column (250 mm × 4.6 mm, 5 μm particle size), n-hexane/isopropyl alcohol/acetic acid (99.05:0.85:0.1, *v*/*v*/*v*) as mobile phase with the flow rate of 1 mL/min at an excitation wavelength of 290 nm and emission wavelength of 330 nm, and compared each tocochromanol monomer in the sample with eight external standards (α, β, γ, δ-tocopherol and α, β, γ, δ-tocotrienol) according to the retention time. Tocopherols and tocotrienols were purchased from Wako Pure Chemical Industries (Tokyo, Japan) and Chromadex Ltd. (Irvine, CA, USA), respectively. Data were expressed as micrograms per gram of sample dry weight (μg/g DW) and were performed as mean ± standard deviation (*n* = 3).

### 2.3. Extraction and Determination of Chlorophyll

Since tocochromanol is the same as the precursor of chlorophyll, this study explores the metabolic changes of tocochromanol by measuring the changes in chlorophyll content. Chlorophyll was extracted and determined as described previously [19], which was modified a little. A 0.5 g sample was mixed with 4 mL of acetone and extracted by ultrasonic for 30 min. The mixture was centrifuged at 5000 rpm for 15 min at 4 °C and the supernatant was obtained. The absorbance was measured at 646 nm and 663 nm, respectively. The contents of chlorophyll a, chlorophyll b and total chlorophyll were calculated according to Equations (1)–(3), respectively. Data were expressed as milligrams per gram of sample dry weight (mg/g DW) and was performed as mean ± standard deviation (*n* = 3).

C_a_ (Chlorophyll a, mg/L) = 12.7A_663nm_ − 2.69A_646nm_ (1)

C_b_ (Chlorophyll b, mg/L) = 22.9A_646nm_ − 4.68A_663nm_ (2)

C_T_ (Total chlorophyll, mg/L) = C_a_ + C_b_ (3)

Chlorophyll content (mg/g) = C (mg/L) * acetone volume (L)/sample weigh (g)(4)

### 2.4. RNA Extraction, cDNA Synthesis and RT-PCR Analysis

Total RNA from the developing pomelos was isolated using Plant RNA Kit (Tiangen Biotech, Beijing, China), and cDNA synthesis was performed by FastKing RT kit with gDNase (Tiangen Biotech, Beijing, China). Quantitative real-time PCR analysis was carried out by Talent qPCR PreMix with SYBR Green (Tiangen Biotech, Beijing, China) and completed by Ligh*TC*ycler^®^ 480 Real-Time PCR System (F. Hoffmann-La Roche Ltd., Basel, Switzerland). *ACTIN* (*Citrus clementina*) was selected as the reference gene. All primer sequences involved in the study are listed in Table 1. Relative expression of each gene was calculated by the 2^−ΔΔCt^ method. Data were expressed as mean ± SE (*n* = 3).

### 2.5. Statistical Analysis

The differences among data of three biological replicates from different stages were analyzed by Duncan’s and Pearson’s multiple comparison test (*p* < 0.05) with SPSS software 26.0 (IBM Inc., Armonk, NY, USA). Data analysis was completed by Origin Pro 2021(Origin Lab Corporation, Northampton, MA, USA). All measurements were carried out in triplicates and the data were expressed as means ± SD (*n* = 3).

## 3. Results

### 3.1. Dynamic Changes of Chlorophyll Profiles during Early Fruit Development

**Figure 2:**
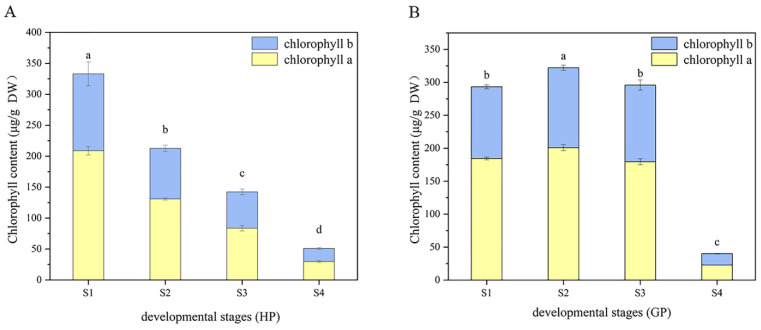
Dynamic changes of chlorophyll during fruit development in the two types of pomelos. (**A**) Honey pomelo; (**B**) Golden pomelo. Different letters indicate significant differences between values (*p* < 0.05).

The changes of chlorophyll composition in different periods were shown in Figure 2. It could be found that the content of chlorophyll a was always higher than chlorophyll b in the four-growth process of two pomelo genotypes. In honey pomelo, the total chlorophyll content was the highest (333 ± 17 μg/g DW) at S1, and then decreased significantly to the lowest (51 ± 3 μg/g DW) at the following three stages, only 15.33% of the initial content at S4. The change trends of chlorophyll a and b were similar to that of total chlorophyll. The maximum value of chlorophyll a was 208.77 ± 6.86 μg/g DW at S1, and the minimum value (30 ± 2 μg/g DW) was only 14.25% of the initial value at S4. The maximum value of chlorophyll b was 124.26 ± 19.28 μg/g DW at S1, and the minimum value (21 ± 1 μg/g DW) was only 17.15% of the initial value at S4.

In golden pomelo, the total chlorophyll content reached the peak value (322 ± 4 μg/g DW) at S2 but decreased slowly at S3 and rapidly at S4. The minimum value (40.02 ± 0.70 μg/g DW) was only 12.42% of the maximum value. The change trends of chlorophyll a and b were similar to that of total chlorophyll. The maximum value of chlorophyll a was 200.84 ± 4.56 μg/g DW at S2, and the minimum value (22.79 ± 0.20 μg/g DW) was only 11.35% of the maximum value at S4. The maximum value of chlorophyll b was 121 ± 4 μg/g DW at S2, and the minimum value (17.23 ± 0.52 μg/g DW) was only 14.21% of the maximum value at S4.

### 3.2. Dynamic Changes of Tocochromanol Profiles during Early Fruit Development

**Figure 3:**
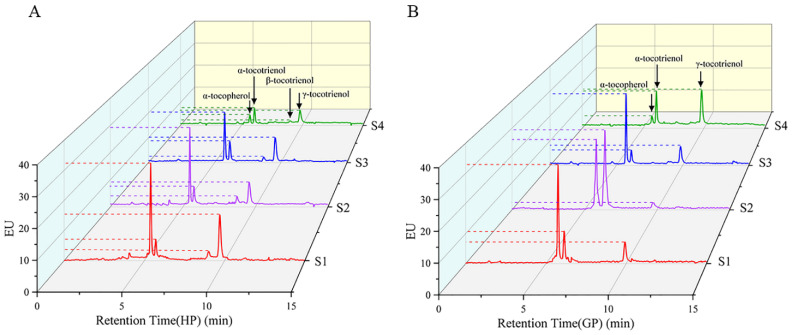
HPLC profiles of tocochromanol in young pomelo fruits. (**A**) Honey pomelo; (**B**) Golden pomelo.

**Figure 4:**
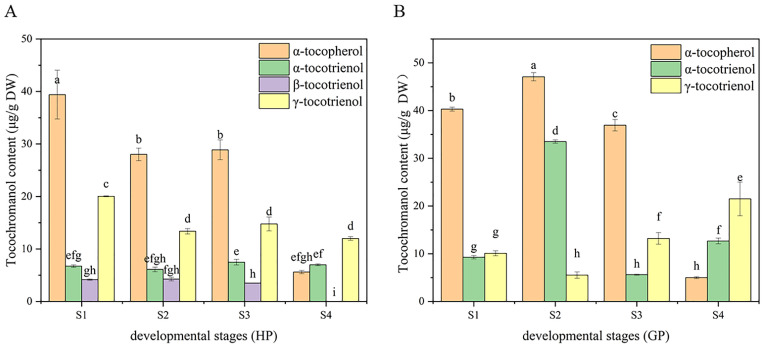
Dynamic changes of tocochromanol during fruit development in the two types of pomelos. (**A**) Honey pomelo; (**B**) Golden pomelo. Different letters indicate significant differences between values (*p* < 0.05).

The tocochromanol in pomelo was determined by HPLC-FL. α-tocopherol, α-tocotrienol and γ-tocotrienol were detected in both pomelo genotypes, while β-tocotrienol was only detected in honey pomelo. The changes in chromatograms during early fruit development are shown in Figure 3. As shown in Figure 4, the highest tocochromanol content (70 ± 5 μg/g DW) in honey pomelo was at S1, then decreased at the S2 stage and slightly increased at the S3 stage. However, the total tocochromanol content sharply decreased to the lowest (24.57 ± 0.41 μg/g DW) at S4, 35% of the initial content. The content of total tocochromanol in golden pomelo increased to the maximum (86.10 ± 0.18 μg/g DW) at the S2 stage compared with the S1 stage (59.64 ± 0.14μg/g DW). It decreased at the S3 stage (56 ± 2 μg/g DW) and the content continued to decrease to the lowest (39 ± 3 μg/g DW) at the S4 stage. At the four stages, the total average tocochromanol contents of honey pomelo and golden pomelo were 50 ± 19 μg/g DW and 61 ± 19 μg/g DW, respectively. Overall, the total tocochromanol content of honey pomelo is lower than that of golden pomelo. At the four developmental stages of honey pomelo, the total content of tocochromanol decreased slowly at first and then decreased sharply, whereas the total content of tocochromanol in golden pomelo increased rapidly at first then decreased sharply, and finally decreased slowly (Figure 4).

In honey pomelo, the total content of tocopherol in the first three periods was higher than that of tocotrienol, but the total content of tocotrienol was 3.39 times that of tocopherol at S4. The content of α-tocopherol was the highest, followed by γ-tocotrienol, and β-tocotrienol was the lowest in honey pomelo. The variation trend of α-tocopherol was similar to the total tocochromanol during the fruits’ early development of honey pomelo. The contribution rate of α-tocopherol to total tocochromanol exceeded 50% (52.88% to 56.02%) in the first three periods, and the content of α-tocopherol was the highest at S1 stage (39 ± 5 μg/g DW). Although the total content of tocotrienol showed a downward trend, the contribution rate showed an upward trend, and the contribution rate reached a peak at S4 (77.22%). The tocochromanol contribution rate of γ-tocotrienol to the growth and development of honey pomelo was 25.85% to 28.49% in the first three periods, but it was 48.78% at S4. Although there was no significant difference in the content of α-tocotrienol in four periods of pomelo development, the contribution rate of α-tocotrienol to the first three periods was 9.57% to 13.69, but significantly increased to 26.14% at S4. The contribution rate of β-tocotrienol to the growth and development of honey pomelo was the lowest (5.91% to 8.23%) at the first three stages and was not detected in S4. In golden pomelo, the changing trend of total tocopherol content was similar to that of honey pomelo, but the total tocotrienol content was 6.85 times that of total tocopherol content at S4. α-tocopherol contributed the most (67.54%) at S1. However, the contribution rate gradually decreased during early fruit development and decreased significantly (12.74%) at S4. The total content of tocotrienol reached the peak at S2 (39.02 ± 0.86 μg/g DW), but decreased to the lowest at the S3 stage (19 ± 1 μg/g DW), and increased again at the S4 stage (34 ± 3 μg/g DW) with the maximum contribution rate (87.26%). The initial content of α-tocotrienol was the lowest (9.27 ± 0.35 μg/g DW), the contribution rate of α-tocotrienol to tocochromanol increased significantly at S2, increased by 38.24% to reach the peak (33.51 ± 0.35 μg/g DW), and the contribution rate of tocochromanol was 38.92%. However, the content (5.61 ± 0.12 μg/g DW) and contribution rate (10.06%) decreased to the lowest at S3 and rebounded at S4. The content of γ-tocotrienol was lower in the first two periods, especially at S2 (5.51 ± 0.68 μg/g DW), but the contribution rate of tocochromanol was the highest at S4 (54.91%). It can be found that the pattern of tocochromanol content was different in the four developmental stages of the two pomelo genotypes. However, there was a similar pattern in both: tocochromanol and α-tocopherol content were higher in the first three periods, but they both decreased sharply at the S4 stage.

### 3.3. Differential Genes Expression in Tocochromanol Biosynthesis Pathway

**Figure 5:**
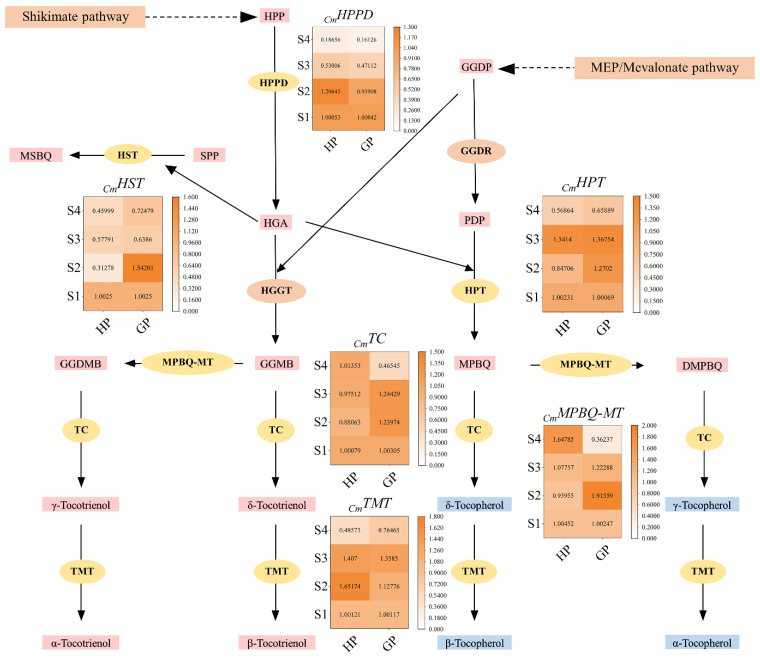
Tocochromanol biosynthesis pathways and related gene expression levels during early fruit development. Values are showed as means ± SD (*n* = 3). HPP, 4-hydroxyphenylpyruvate; HGA, homogentisic acid; HPT, homogentisate phytyltransferase; HST, homogentisate solanesyl transferase; HPPD, 4-hydroxyphenyl-pruvate dioxygenase; MPBQ-MT, 2-methyl-6-phytylbenzoquinol methyltransferase; TMT, tocopherol methyltransferase; TC, tocopherol cyclase. MPBQ, 2-methyl-6-phytyl-1,4-benzoquinol; DMPBQ, 2,3-dimethyl-5-phytylbenzoquinol; MEP, methylerythritol phosphate. PDP, phytyl diphosphate; GGDP, geranylgeranyl diphosphate; SPP, solanesyl diphosphate.

The relative expression levels of selected genes in tocochromanol biosynthesis pathway were shown in Figure 5. Homogentisate phytyl transferase (HPPD) catalyzes HPP to homogentisic acid (HGA), which is the first enzyme in the tocochromanol biosynthesis pathway. The results showed that the encoding gene of HPPD was up-regulated at S2, but significantly down-regulated at S3 and S4 in honey pomelo. In contrast, the expression of *_Cm_HPPD* in golden pomelo was continuously down-regulated in four periods and significantly down-regulated at S3 and S4. Overall, the expression of *_Cm_HPPD* was both higher at S1 and S2 stages but lower at the latter two stages. In contrast, the average expression of *_Cm_HPPD* in honey pomelo was higher than that in golden pomelo.

Homogentisate solanesyl transferase (HST) catalyzes solanesyl diphosphate (SPP) and HGA to 2-methyl-6-solanesyl-1,4-benzoquinol (MSBQ) in tocochromanol biosynthesis pathway. The expression of *_Cm_HST* in honey pomelo was higher at S1, but it was significantly down-regulated to the lowest at S2 (only 31.2% of the initial period), and it was up-regulated at S3 but down-regulated again at S4. In golden pomelo, *_Cm_HST* was significantly up-regulated at S2, but down-regulated in S3 and slightly up-regulated at S4. Overall, the average expression of *_Cm_HST* in golden pomelo was higher than that in honey pomelo.

Homogentisate phytyl transferase (HPT) catalyzes phytyl diphosphate (PDP) and HGA to 2-methyl-6-phytylbenzoquinol (MPBQ) in tocochromanol biosynthesis pathway. The expression of *_Cm_HPT* was down-regulated at the S2 stage, up-regulated at the S3 stage, and down-regulated again at S4 in honey golden. The expression of *_Cm_HPT* increased at S2 and S3 and down-regulated at S4. In general, the expression levels of *_Cm_HPT* in the two golden genotypes at S2, S3 and S4 stages were higher, but they were lower at S4. Moreover, the average expression of *_Cm_HPT* in golden pomelo was higher than that in honey pomelo, just like *_Cm_HST*.

2-methyl-6-phytylbenzoquinol methyltransferase (MPBQ-MT) catalyzes 6-geranylgeranyl-2-methylbenzene-1,4-diol (GGMB) to geranylgeranyl-2,3-dimethylbenzene-1,4-diol (GGDMB) in tocochromanol biosynthesis pathway. The expression of *_Cm_MPBQ-MT* in pomelo was slightly down-regulated at S2, but it was continuously up-regulated at S3 and S4 in honey pomelo. The expression of S2 reached the peak in golden pomelo, but down-regulated continuously at S3 and S4. In contrast, the average expression of *_Cm_MPBQ-MT* in honey pomelo was lower than that in golden pomelo at the first three stages. However, the expression of *_Cm_MPBQ-MT* was the highest in honey pomelo and lowest in golden pomelo at S4, which made the average expression of honey pomelo higher than golden pomelo at four stages.

Tocopherol cyclase (TC) in tocochromanol biosynthesis pathway catalyzes GGDMB to γ-tocotrienol, catalyzes GGMB to δ-tocotrienol, catalyzes 2-methyl-6-phytyl-1,4-benzoquinol (MPBQ) to δ-tocopherol, and catalyzes DMPBQ to γ-tocopherol. The expression levels of *_Cm_TC* in honey pomelo changed little and were all higher at the four stages. *_Cm_TC* slightly down-regulated at S2 and continuously up-regulated at S3 and S4. In golden pomelo, *_Cm_TC* was highly expressed in the first three periods and continued to up-regulated at S2 and S3, but S4 content was only 46.4% of the initial. Overall, the average expression of *_Cm_TC* in honey pomelo was slightly lower than that in golden pomelo.

Tocopherol methyltransferase (TMT) is the last enzyme in tocochromanol biosynthesis pathway, which catalyzes γ-tocotrienol to α-tocotrienol, catalyzes δ-tocotrienol to β-tocotrienol, catalyzes δ-tocopherol to β-tocopherol, and catalyzes γ-tocopherol to α-tocopherol. *_Cm_TMT* was highly expressed in the first three periods in honey pomelo, up to peak at S2, but slightly down-regulated at S3 and significantly decreased in S4. In contrast, the expression of *_Cm_TMT* in golden pomelo was continuously up-regulated in the first three periods, and significantly down-regulated at S4. Overall, the average expression of *_Cm_TMT* in golden pomelo was higher than that in honey pomelo.

### 3.4. Correlations between Compositions and Gene Expressions

**Figure 6:**
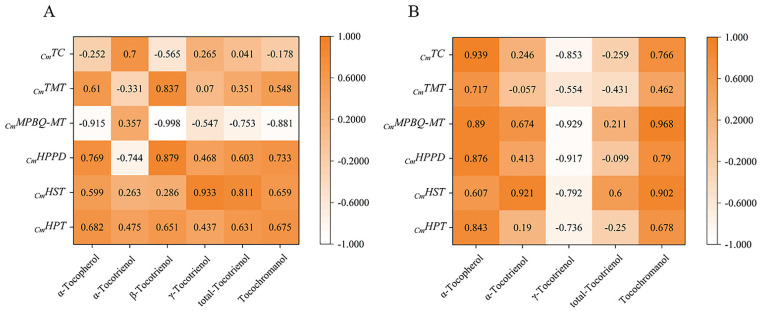
Pearson correlations between tocochromanol (**A**,**B**) compositions contents and their gene expression levels. (**A**) The correlations of honey pomelo. (**B**) The correlations of golden pomelo.

**Figure 7:**
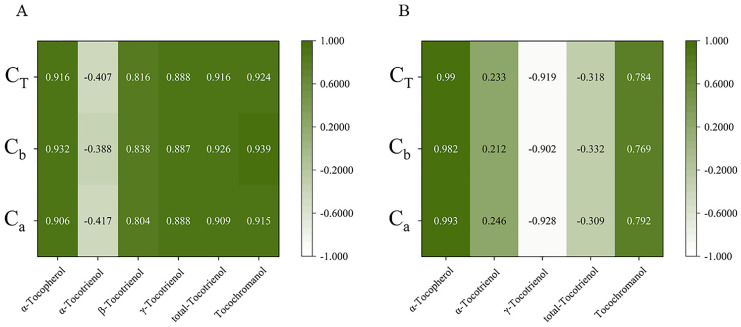
Pearson correlations between tocochromanol (**A**,**B**) compositions content and chlorophyll compositions content. (**A**) The correlations of honey pomelo. (**B**) The correlations of golden pomelo. C_T_, total chlorophylls; C_b_, chlorophyll b; C_a_, chlorophyll a.

The relationship between expression and content of different genes in the four early developmental stages was analyzed by Duncan and Pearson multiple comparison test (*p* < 0.05) using SPSS software 26.0. As shown in Figure 6, the relative expressions of most genes were positively correlated with tocochromanol contents during early fruit development. In golden pomelo, all genes except *_Cm_TMT* were positively correlated with α-tocotrienol, but only *_Cm_MPBQ-MT* and *_Cm_HST* had higher correlation values. However, all genes were negatively correlated with γ-tocotrienol. All genes in Figure 6 were significantly positively correlated with α-tocopherol and total tocochromanols, but only *_Cm_MPBQ-MT* and *_Cm_HST* were positive correlated with total tocotrienols. In honey pomelo, the expression of *_Cm_HPT* was positively correlated with the four measured tocochromanols. Although the relative expression level of *_Cm_HST* was also positively correlated with four tocochromanol components, the correlation with α-tocotrienol and β-tocotrienol was lower. In addition to α-tocotrienol, *_Cm_HPPD* has a significant positive correlation with other tocochromanol. Overall, *_Cm_HPPD*, *_Cm_HST* and *_Cm_HPT* have a higher correlation with α-tocopherol and total tocotrienols. As shown in Figure 7, all tocochromanols except α-tocotrienol showed a good correlation with chlorophylls in honey pomelo. In golden pomelo, α-tocopherol and total tocochromanols showed a good correlation with chlorophylls.

## 4. Discussion

### 4.1. Variations in Tocochromanol Accumulation and Gene Expression

Tocochromanol is widely distributed in plants. The type and content of tocopherols varied considerably among plants. In addition, different developmental stages also affect the content of tocochromanol [20]. To better understand the molecular mechanisms behind the changes in the content of each tocochromanol component during the development of young pomelo fruits, we selected six key coding genes in the relevant biosynthetic pathway (*_Cm_HPT*, *_Cm_TC*, *_Cm_TMT*, *_Cm_HST*, *_Cm_MPBQ-MT* and *_Cm_HPPD*) and investigated the expression of the relevant genes in young pomelo fruits of two genotypes during four developmental periods by using RT-qPCR. In general, the relative expression levels of the genes differed during fruit development (Figure 5).

All or part of tocochromanol is synthesized in the plastidic isoprenoid biosynthetic pathway, specifically the MEP pathway, which produces the key intermediate for synthesizing tocochromanol, geranylgeranyl diphosphate (GGDP) [5]. Fluxes of the MEP pathway are involved not only in the biosynthesis of tocochromanol but also in the synthesis of other metabolites, such as gibberellin and chlorophyll [21]. This may also be one of the reasons for the gradual decrease in tocochromanol in young pomelo fruits during development. Tocopherol cyclase (TC) and tocopherol methyltransferase (TMT) determine the type of tocopherols or tocotrienols accumulated in plant tissues, and variations in their expression patterns may be partly responsible for the different tocochromanol composition in different genotypes of pomelo. For example, TC expression in golden pomelo was increased by the accumulation of α-tocopherol and α-tocotrienol at S2. However, S3 showed a decreasing trend in α-tocopherol and α-tocotrienol without significant changes in *_Cm_TC* expression, and *_Cm_TMT* showed a similar situation. This suggested the involvement of other regulatory mechanisms, such as post-transcriptional regulation, and further studies would be required. In addition, in golden pomelo, α-tocopherol was the predominant form during the first three periods of sustained up-regulation of *_Cm_TMT*, suggesting that *_Cm_TMT* might have a substrate preference for α-tocopherol over tocotrienols, and a similar phenomenon was found during rice seed development [22].

Although the accumulation of tocochromanol in seeds was found to be essential for seed germination, little is known about the pattern of tocochromanol production and accumulation during fruit development [20]. To the best of our knowledge, most studies have shown an increasing trend of tocochromanol content during seed germination, including soybean [23], maize seeds [24] and sesame seeds [25]. However, Kim et al. [26] reported that lower tocochromanol levels were observed in germinated rice, which was similar to our study results. Our study showed an overall decreasing trend in the total tocochromanol content of young pomelo fruits during development. This phenomenon suggested that the rate of tocochromanol consumption exceeded the production rate in young pomelo fruits during early development, resulting in a significant decrease in tocochromanol levels. However, the cause of this situation has not known at present. We observed a significant increase (*p* < 0.05) in α-tocopherol content during the second stage of golden pomelo development. This might be caused by chlorophyll degradation to produce prerequisite substances for tocopherol synthesis (tocopherols are synthesized by photosynthesizing organisms) [20]. On the other hand, our study showed that tocotrienols were the major tocochromanol in young pomelo fruits at S4 (87.26% in golden pomelo and 77.22% in honey pomelo). Therefore, tocotrienols might play an important role in promoting fruit ripening at a later stage.

Many researchers have studied the expression of 4-hydroxyphenylpropanoic acid dioxygenase (HPPD) and have shown that overexpression of *_Cm_HPPD* may induce an increase in tocochromanol in barley, tobacco seeds, and potato tubers [27]. However, *_Cm_HPPD* overexpression in Arabidopsis increased enzyme activity but only increased tocopherol by approximately 30% [28], and our study found that *_Cm_HPPD* did not correlate particularly well with tocopherol component during early fruit development(*r* < 0.9). It is hypothesized that *_Cm_HPPD* has a limited effect on tocopherol.

### 4.2. Changes of Tocochromanol Composition and Contents during Fruit Development

Tocochromanol was shown to be beneficial for animal reproduction and may also promote human health by inhibiting lung cancer, slowing brain aging, inhibiting cholesterol production, and reducing the risk of Alzheimer’s disease [29]. In this study, HPLC was used to separate and determine the composition and content of tocochromanol in the samples. Figure 3 shows chromatograms of young pomelo fruit extracts, where one tocopherol and two tocotrienols were identified from both species during fruit development, while β-tocotrienols were also found in honey pomelo. Previous studies have found that grape (non-climacteric) has a progressive decrease in tocopherol content during seed development [30]. However, the opposite pattern was observed in respiratory climacteric fruits such as mango [31] and tomato [32]. In contrast, in the present experiment, total tocochromanol content in honey pomelo showed an overall decreasing trend at the beginning of fruit development (S1 to S4), maintaining a stable content during S2 and S3, and finally decreasing rapidly to 24.57 ± 0.41 μg/g DW at S4. In contrast, total tocochromanol content in golden pomelo showed a different trend, first peaking during S2 and then continuing to decrease, reaching a minimum value during S4 (39 ± 3 μg/g DW). The pomelo also belongs to the non-respiratory leap type such as grape, suggesting that ethylene is a regulatory molecule for tocochromanol biosynthesis. This hypothesis needs further investigation. Both α-tocopherol and tocotrienol contents in honey pomelo reached a maximum at S1, while golden pomelo reached a maximum at S2. Tocopherol content was higher than tocotrienols during the first three periods of ripening, which was the case in both genotypes. The mean values of total tocochromanol in the four developmental periods of the fruit were 50 ± 19 μg/g DW and 60 ± 19 μg/g DW for honey pomelo and golden pomelo, respectively, so the consumption of foods or supplements containing extracts made from these young fruits can easily meet the daily intake of humans (15 mg/day) [19].

According to previous studies, α-tocopherol is the most abundant form of all tocopherols compared to all other tocopherols and tocotrienols [33]. A related study found that α-tocopherol accounted for 90.9% of all other tocopherols in tomato fruit [32], in addition, an assay of α- and γ-tocopherol content in commercial hawthorn showed that α-tocopherol was the dominant compound in all samples, with an average of 66.9% [34]. Our study found that both genotypes of pomelo had a high content of α-tocopherol, with the highest content of 47.08 ± 0.85 μg/g DW in golden pomelo, with the highest percentage of 67.54%. The highest content of 39 ± 5 μg/g DW was found in honey pomelo, with the highest percentage of 56.02%.

### 4.3. Potential Interrelationships of Tocochromanol and Chlorophyll in Pomelo during Fruit Development

There is an indirect link between the metabolism of substances in plants, with tocochromanol biosynthesis occurring in the inner layers of the plastids, and several studies have shown a strong correlation between chlorophyll loss and tocopherol production [35]. α-Tocopherol, the primary form of tocopherols in photosynthetic tissues, regulates lipid peroxidation in chloroplasts and also has antioxidant effects in some fruits [35]. Chlorophyll degradation occurs in plant responses to environmental stresses and during fruit ripening, which is consistent with our results. The study has shown that chlorophyll degradation and tocopherol production are strongly correlated, that PDP is associated with chlorophyll degradation through the phytol phenolic cycle pathway. That chlorophyll synthase and geranylgeranyl diphosphate reductase (GGDR) are also involved in tocochromanol biosynthesis [35].

Most previous studies have focused on the synthetic pathway of tocopherols and less on the relationship between tocochromanol and chlorophyll metabolism. Our study attempted to reveal the potential interdependence between tocochromanol and chlorophyll metabolism. There was a significant correlation between chlorophyll and α-tocopherol (*r* > 0.9) and a significant negative correlation with γ-tocotrienols (*r* < −0.9) during young fruit development in golden pomelo. In contrast, in honey pomelo, only α-tocotrienol showed a negative correlation with chlorophyll content, while all other components showed a positive correlation, especially α-tocopherol (*r* > 0.9). Both chlorophyll and total tocochromanol decreased with fruit development, probably due to a decrease in precursors such as PDP or the synthesis of other substances to promote fruit ripening. Higher levels of tocopherols were detected in tomato leaves, which was consistent with the hypothesis that it was involved in protecting photosynthetic bodies in chloroplasts [36]. Therefore, we speculated that it was also possible that the decrease in chlorophyll content, which needs to be protected with fruit development, led to a concomitant decrease in tocochromanol content.

## 5. Conclusions

In summary, this study investigated tocochromanol metabolism and transcript levels in young pomelo fruits during early development, revealing the pattern of variations in tocochromano and chlorophyll content in different genotypes of pomelo during early fruit development, partly elucidating the molecular mechanisms behind these changes by studying the key encoding genes in the biosynthetic pathway. In golden pomelo, most of the components accumulated substantially in the S2 period, while γ-tocotrienol content decreased but continued to accumulate during the latter two periods. In contrast, the content of all tocochromanol components except α-tocotrienol showed an overall decreasing trend in honey pomelo. The expression patterns of relevant genes in the biosynthetic pathway partially explained the changes in tocochromanol during the development of young pomelo fruit, where higher expression levels of key genes would lead to higher total tocochromanol accumulation in young pomelo fruit. This study found that the first stage of honey pomelo and the second stage of golden pomelo were the best extraction period. Therefore, the honey pomelo at S1 and the golden pomelo at S2 may be important sources for industrial extraction of tocochromanol, and putting the tocochromanol into the production of natural additives will produce more economic benefits in the food, cosmetics and pharmaceutical industries.

## Figures and Tables

**Table 1:** Primer sequences of related genes for Real-Time PCR.

Gene Name	Gene ID	Prime Direction	Primer Sequence 5′-3′
*_Cm_HPPD*	LOC18044823	Forward	TGTTGAAGTTGAAGACGCCG
		Reverse	AATCTCATCCGTCGGTTCGA
*_Cm_HPT*	LOC18055996	Forward	GTGTCAGTTGCTCTCCTTGC
		Reverse	AGCACACCAGTCTTGAAGGA
*_Cm_HST*	LOC18039406	Forward	CTAAGGCCACACACAATCCG
		Reverse	TCCCGCAGCTATTGGTAAGT
*_Cm_MPBQ-MT*	LOC18048001	Forward	AGAGACGATGCACTAGAGCC
		Reverse	AGTGGCTCCTTTTGCTTAGC
*_Cm_TC*	LOC18055331	Forward	TGGGAATACAGTACTCGGCC
		Reverse	TTCGCCATCCCACTCTATCC
*_Cm_TMT*	LOC18045307	Forward	TGTTGTGGATGTTGGCTGTG
		Reverse	TGTCAGGCATGTGTTCTCCA
*_Cm_ACTIN*	LOC18038212	Forward	GCTATCCAGGCTGTGCTTTC
		Reverse	AACAATTTCCCGCTCAGCAG

## Data Availability

The data presented in this study are available in the article.
